# Validity and reliability evidence for the Behavioral Regulation in Sport Questionnaire with Romanian professional athletes

**DOI:** 10.7717/peerj.12803

**Published:** 2022-01-11

**Authors:** Cristina Ioana Alexe, Dan Iulian Alexe, Gabriel Mareş, Dragoş Ioan Tohănean, Ioan Turcu, Rafael Burgueño

**Affiliations:** 1 Department of Physical Education and Sports Performance, Faculty of Movement, Sports and Health Sciences, Vasile Alecsandri University of Bacau, Bacau, Romania; 2 Department of Physical and Occupational Therapy, Faculty of Movement, Sports and Health Sciences, Vasile Alecsandri University of Bacau, Bacau, Romania; 3 Department of Motric Performance, The Faculty of Physical Education and Mountain Sports, Transilvania University of Braşov, Braşov, Romania; 4 Health Research Center and Department of Education, Universidad de Almería, Almería, Spain

**Keywords:** Self-determined motivation, Autonomous motivation, Controlled motivation, Sportspeople, Psychometric properties

## Abstract

**Background:**

Despite the importance attributed to athletes’ motivation in sports performance and well-being; no measures of motivation toward sport were found in the Romanian sport context.

**Objective:**

Grounded in self-determination theory, this research aimed to adapt and to gather validity and reliability evidence supporting the use of the Behavioral Regulation in Sport Questionnaire (BRSQ) in the Romanian sport domain.

**Method:**

The participants were 596 Romanian professional athletes (age: *M* = 22.91, *SD* = 5.84; sports experience: *M* = 11.14, *SD* = 5.03), who 273 practiced individual sports and 323 team sports. They completed an online questionnaire survey assessing their perception of behavioral regulation, resilience and burnout in sport.

**Results:**

Confirmatory factor analysis supported the six-factor correlated model, which was invariant across age and sport. Correlations among latent factors configured a *simplex* structure, underpinning the self-determination *continuum*. Average variance extracted values from .50 to .70 endorsed convergent validity. Scores for heterotrait-monotrait ratio of correlations as high as .88, as well as 95% confidence intervals of each interfactor correlation that did not include 1.00 supported discriminant validity. Values over .70 for Cronbach’s alpha, McDonald’s omega and Raykov’s coefficients showed a good level of reliability for each factor. Linear regression analysis revealed that while intrinsic motivation, integrated regulation and identified regulation positively predicted resilience, introjected regulation, external regulation and amotivation positively predicted burnout.

**Conclusions:**

The BRSQ is shown to be a valid and reliable measure of the six types of behavioral regulation in the Romanian sport context.

## Introduction

Motivation plays a key role in sport performance and well-being of athletes (Clancy et al., 2016; Gillet & Vallerand, 2016; Badau & Badau, 2018; Jankauskiene et al., 2019; Gherghel et al., 2021). Unlike classical motivational theories that exclusively conceived motivation in quantitative terms (see Eccles & Wigfield, 2002), self-determination theory (SDT) conceptualizes motivation both from a quantitative and qualitative perspective (Ryan & Deci, 2020). To illustrate, an athlete with a great quantity of motivation might not undertake the target behavior, if the motivation implied were of a low quality (Gustafsson et al., 2018). This distinctiveness in conceptualizing motivation has made that SDT become the predominant theoretical framework for the study of motivation in the sport context (Clancy et al., 2016). To measure motivation toward sport, several SDT-based instruments were developed at the international level over the last decades (Clancy, Herring & Campbell, 2017); however, there are no scales to date that assess motivation in the Romanian sport setting. Taking into consideration that the Behavioral Regulation in Sport Questionnaire (BRSQ; Lonsdale, Hodge & Rose, 2008) constitutes the most broadly used instrument in judging the athletes’ perception of motivation toward sport (Clancy, Herring & Campbell, 2017; Rodrigues et al., 2020), the purpose of this study was to adapt and to examine the psychometric properties of the BRSQ using a sample of Romanian professional athletes.

### Self-determination theory

SDT is a macro-theory of motivation, personality and wellness resting on organismic (*i.e.,* individuals are hypothesized to be oriented toward growth) and dialectic (*i.e.,* growth occurs through environmental interactions) assumptions (Deci & Ryan, 1985). SDT proposes a multidimensional conceptualization for motivation holding the idea that distinct types or qualities of motivation will yield differentiated affective, cognitive and behavioral consequences for individuals (Ryan & Deci, 2020). SDT distinguishes between three types of motivation that fall along a self-determination *continuum*, reflecting to what extent the behavior is intentionally and autonomously undertaken and consistent with the individual’s own values and interests (Ryan & Deci, 2020; Ryan et al., 2021).

At an extreme of the self-determination *continuum* stands intrinsic motivation, conceptualized as undertaking a behavior for the inherent interest, pleasure and enjoyment, as well as curiosity and the search for new challenges. In the central part of this *continuum* lays on extrinsic motivation, which refers to the adoption of a behavior as a means to an end. Given its instrumental nature, extrinsic motivation contemplates four types of behavioral regulation depending on the variability in the internalization process (*i.e.,* “the active assimilation of a behavioral regulation that are originally alien to the self”; Deci & Ryan, 1985). External regulation describes the full absence of behavioral internalization, in which behavior is undertaken to fulfill external demands such as obtaining social or material rewards or avoiding punishments from an external source. Introjected regulation expresses a partial degree of behavioral internalization, in which behavior is performed by self-imposed pressures and internal contingencies in order to gain self-worth or to avoid feelings of shame and guilt. Identified regulation represents an almost complete degree of behavioral internalization, in which behavior is undertaken for its personal value and the recognition of the benefits associated with its adoption. Integrated regulation reflects the highest degree of behavioral internalization, in which behavior is adopted by being perceived as part of the identity of the person. At the opposite extreme of the self-determination *continuum* and in contrast to intrinsic and extrinsic motivation, one finds amotivation, defined as the total absence of self-determination and intention with regard with the desired behavior.

Although the classical distinction between intrinsic motivation, extrinsic motivation and amotivation continues to be broadly recognized and accepted in the SDT-based research, the identification that some forms of extrinsic motivation are relatively autonomous has supposed a conceptual shift in the SDT framework (Vansteenkiste, Niemiec & Soenens, 2010). Thus, this paradigm has been replaced by a distinction between autonomous motivation (including intrinsic motivation, integrated regulation and identified regulation), controlled motivation (including introjected and external regulation) and amotivation (Vansteenkiste, Niemiec & Soenens, 2010; Ryan et al., 2021).

In this regard, the SDT postulates that autonomous forms of motivation would lead to adaptive affective behavioral and cognitive outcomes (Ryan & Deci, 2020; Ryan et al., 2021) such as performance (Koka et al., 2020), commitment (Pulido et al., 2018), intention to continue the sports practice (Monteiro et al., 2018) and resilience (Trigueros et al., 2020) among athletes. Particularly, from a theoretical viewpoint, the relationship between autonomous forms of motivation and resilience is argued in the fact that autonomously motivated athletes often tended to improve their skills and overcome challenges regardless of unfavorable and stressful events, promoting a resilient behavior (Trigueros et al., 2020). In contrast, the SDT posits that controlled forms of motivation and amotivation would tend to yield maladaptive outcomes (Ryan & Deci, 2020; Ryan et al., 2021) such as anxiety and disengagement (Martinent et al., 2018), performance failure (Gómez-López, Courel-Ibáñez & Granero-Gallegos, 2021) and burnout (Lonsdale & Hodge, 2011; Li et al., 2013) in the sport context. Particularly, athletes, guided by controlled reasons or amotivated, were prone to suffer from chronic psychological syndromes such as emotional and physical exhaustion, reduced sense of achievement and sport devaluation, which are strongly associated with burnout (Li et al., 2013).

### Measuring motivation in sport from self-determination theory

Notwithstanding the importance attributed to motivation for the optimal development of adaptive performance-related outcomes in sport (Clancy et al., 2016; Gillet & Vallerand, 2016); to the best of our knowledge, there is no evidence that SDT-based measures have been utilized to assess the athletes’ perception of motivation toward sport in the Romanian domain. This has hindered the full understanding of the motivational dynamics experienced by Romanian athletes throughout their season(s) or Olympic cycle(s), in addition to hampering coaches to accurately detect athletes who might be at a motivational risk at any point of the season. In this same vein, the lack of a valid and reliable measures of motivation has impeded the analysis of influence of the motivational process involved in sport both on performance-related outcomes and health among Romanian athletes. Overall, this has hampered to gain insight into the degree of involvement of athletes in their training and/or competition, which made it impossible to build focus, intensity, ability to follow the competitional and training goals (Clancy, Herring & Campbell, 2017). Besides, the absence of measures of motivation in Romania has limited the international comparison between Romanian athletes and ones from another countries in terms of motivational processes. Lonsdale, Hodge & Rose (2008) developed for international use the BRSQ to judge the competitive athletes’ perception of motivation in sport, which has become the most commonly used tool for its measurement in the sport domain (Clancy, Herring & Campbell, 2017; Rodrigues et al., 2020). According to Lonsdale et al. (2014), this instrument was created in an attempt to minimize the psychometric problems detected in the previous contextual measures of motivation in sport (*e.g.*, the Sport Motivation Scale, (Pelletier et al., 1995; Pelletier et al., 2013).

The BSRQ was designed to allow for the examination of motivation toward sport by two different versions, denominated as the BRSQ-6 and the BRSQ-8 (Lonsdale, Hodge & Rose, 2008). The distinction among both versions refers to the specific manner to operationalize intrinsic motivation. The BRSQ-6 conceptualizes intrinsic motivation as an unitary construct in accordance with the SDT assumptions (Deci & Ryan, 1985). This version thereby consists of six factors assessing intrinsic motivation, integrated, identified, introjected and external regulation, and amotivation, specifying a 24-item, six-factor model. On the other hand, the BRSQ-8 relied on the tripartite conceptualization suggested by Vallerand & Blais (1987) for intrinsic motivation, differentiating between three specific types of intrinsic motivation (*i.e.,* intrinsic motivation to know, toward accomplishments and to experience stimulation). Hence, this version includes eight factors measuring the three subtypes of intrinsic motivation, the four regulatory forms of extrinsic motivation and amotivation, specifying a 32-item, eight-factor model. Although a small body of research has psychometrically underpinned the BRSQ-8 (Moreno-Murcia et al., 2011; Filippos et al., 2019; Pinto-Guedes, Aparecido-Caus & Lucas-Sofiati, 2019), the previous meta-analysis studies (Chemolli & Gagné, 2014; Howard, Gagné & Bureau, 2017) have unrecommended the consideration of the three subtypes of intrinsic motivation in measures of motivation, including the BRSQ, due to excessively high correlations among them and overlapping confidence intervals.

The previous studies have gathered a consistent basis of evidence in psychometric support for the BRSQ-6 in different contexts using athletes with distinct characteristics such as British young athletes (*χ*^2^ = 557.37; CFI = .95; TLI = .94; SRMR = .06; RMSEA = .06, (Holland et al., 2010)) and recreational dancers (*χ*^2^ = 1027.24, CFI = .96; TLI =.95; RMSEA = .06, (Hancox et al., 2015), Swedish young competitive athletes (*χ*^2^ = 260.60; CFI = .95; TLI = .94; RMSEA = .042, (Stenling et al., 2018), Turkish university athletes (*χ*^2^ = 753.78, CFI = .97, TLI = .97, RMSEA = .057, (Çetinkaya & Mutluer, 2018), French young competitive athletes (*χ*^2^ = 315.06; CFI = .94; TLI = .93; RMSEA = .047, (Cece et al., 2019), Spanish young athletes (*χ*^2^ = 815.41; CFI = .92; TLI = .92; RMSEA = .06, (Viladrich, Torregrosa & Cruz, 2011), Portuguese athletes (*χ*^2^ = 995.10, CFI = .92; TLI = .90; RMSEA = .066, (Monteiro, Moutão & Cid, 2018), and Brazilian young competitive athletes (*χ*^2^/*df* = 1.87; CFI = .94; RMSEA = .052, (Pinto-Guedes, Aparecido-Caus & Lucas-Sofiati, 2019)). Indeed, the six-factor correlated model psychometrically performed better than alternative two-factor, three-factor, four-factor and five-factor correlated models proposed in previous research (Lonsdale et al., 2014; Hancox et al., 2015; Monteiro, Moutão & Cid, 2018; Rodrigues et al., 2020). Despite the good psychometric performance, previous research also found a lack both of discriminant validity between integrated and identified regulation subscales, and between introjected and external regulation factors (Lonsdale, Hodge & Rose, 2008; Holland et al., 2010), and convergent validity in the integrated regulation factor (Monteiro et al., 2018), as well as a marginal reliability scores in amotivation (Tsitskari et al., 2015). On the other hand, it is noteworthy that evidence was provided in support of measurement invariance across gender and sport (Monteiro et al., 2019), age and performance level (Lonsdale, Hodge & Rose, 2008; Hancox et al., 2015), time (Stenling et al., 2018; Cece et al., 2019), as well as across five European countries (Viladrich et al., 2013).

### The present research

The objective of this research was to adapt and to analyze the psychometric properties of the BRSQ using a sample of Romanian professional athletes. First, validity based on internal structure was analyzed by a confirmatory factor analysis (CFA) approach that tested the robustness of the six-factor correlated model initially proposed by Lonsdale, Hodge & Rose (2008) against different alternative order-primary models identified in the prior research (Lonsdale et al., 2014; Hancox et al., 2015; Monteiro, Moutão & Cid, 2018; Rodrigues et al., 2020) and that could be underpinned by SDT. In addition, we tested the tenability of two hierarchical models to ascertain if the six types of behavioral regulation better adjusted to the classical view (*i.e.,* intrinsic motivation, extrinsic motivation and amotivation) or the new perspective (*i.e.,* autonomous motivation, controlled motivation and amotivation). Once the best-factor model was identified, we extended validity evidence based on internal structure by running two multi-group analyses of invariance across age and sport. Second, convergent and discriminant validity together with reliability were, respectively, inspected. Third, criterion validity was provided by two linear regression analyses. In both analyses, we hypothesized that the three autonomous forms of motivation (*i.e.,* intrinsic motivation, integrated and identified regulation) would positively and significantly predict resilience, while the two controlled forms of motivation (*i.e.,* introjected and external regulation) and amotivation would positively and significantly predict burnout in athletes, consistent with SDT (Ryan & Deci, 2020; Ryan et al., 2021) and previous studies (Li et al., 2013; Clancy et al., 2016; Trigueros et al., 2020).

## Materials and Method

### Participants and setting

The participating sample consisted of 596 Romanian professional athletes (324 men and 272 women) aged between 18 and 52 years (*M* = 22.91, *SD* = 5.84) who competed at the international and national level. They self-reported a sport experience from 7 to 28 years (*M* = 11.14, *SD* = 5.03) at the international or national level. A total of 31 distinct sports were represented with each grouped into individual (*N* = 273, including athletics, gymnastics, rowing, weightlifting, cycling or Olympic shooting) or team sports (*N* = 323, including soccer, basketball, volleyball, rugby, ice hockey or handball).

As a first sept of the research, we estimated a minimum sample of at least 325 participants in accordance with the ratio 5 cases per each statistical parameter specified in the factor model (*i.e.,* 65 parameters) in order to ensure the trustworthiness of the validation results (Kline, 2016). Akin to the method followed in previous studies (Lonsdale, Hodge & Rose, 2008) to recruit and select participants and considering lower response rates on online survey, an e-mail was sent inviting 800 athletes to complete the questionnaire *via* an online survey; 622 athletes (77.75%) responded. Of the totality of 622 athletes, there were 8 (1.29%) cases detected as univariate outliers (*i.e.,*  *Z* < 3.00) and 16 (2.57%) cases as outliers (*i.e.,* Mahalanobis *D*^2^, *p* < .001), which were removed and leading to the final described sample of 596 athletes. The participants in this research had to meet the following inclusion criteria: (a) professional athletes who competed at the international and/or national level, (b) older 18 years old, and (c) signed an informed consent to participate in this study. To complete the online survey, we provided them with instructions and guidelines by explaining them that their participation was fully voluntary and anonymous and there were not right and false responses since we only wanted to know their perception of their training and competitions. The average time estimated for its completion was 20 min. The research was approved by the Ethics Committee of the Vasile Alecsandri University of Bacau (code: 12661/1/27.08.2021).

### Measures

#### Motivation toward sport

To measure athletes’ perceptions of motivation in sport, the Romanian version of the BRSQ (Lonsdale, Hodge & Rose, 2008) was used. The instrument was preceded by the stem “I participate in my sport…” and followed by 24 items (4 items per factor) that measure intrinsic motivation (*e.g.*, “Because I enjoy it”), integrated regulation (*e.g.*, “Because it’s a part of who I am”), identified regulation (*e.g.*, “Because the benefits of my sport are important to me”), introjected regulation (*e.g.*, “Because I would feel ashamed if I quit”), external regulation (*e.g.*, “Because people push me to play”), and amotivation (*e.g.*, “But I question why I continue”). Responses to each item were collected by a 7-point scale ranging from 1 (not at all true) to 7 (very true).

#### Resilience in sport

To measure athletes’ perceptions of resilience in sport, the Romanian version of the Brief Resilience Scale (Smith et al., 2008) was used. The instrument is preceded by the stem “Please indicate the extent to which you agree with each of the following statements” and followed by 6 items (*e.g.*, “I tend to bounce back quickly after hard times”) that assess resilience. Responses to every item were collected by a 5-point Likert-type scale ranging from 1 (strongly disagree) to 5 (strongly agree).

#### Burnout in sport

To assess athletes’ perceptions of burnout in sport, the Romanian version of the Athlete Burnout Measure (Raedeke & Smith, 2001) was used. The instrument is preceded by the stem “In my trainings and competitions…” and followed by 15 items (five per factor) that measure emotional/physical exhaustion. (*e.g.*, “I am exhausted by the mental and physical demands of my sport”), reduced sense of accomplishment (*e.g.*, “I am not performing up to my ability in sport”), sport devaluation (*e.g.*, “I’m not into my sport like I used to be”). Items were responded using a 5-point Likert-type scale ranging from 1 (almost never) to 5 (almost always).

### Design and procedure

As this study aimed at testing the psychometric properties of a measurement instrument, an instrumental design was adopted (Ato, López & Benavente, 2013). The authors received permission to use this instrument from the copyright holders. The BSRQ was adapted to the Romanian context using the guidelines proposed by Bartram et al. (2018). A group of 2 translators translated the instrument into Romanian and, subsequently, a distinct group of 2 translator translated the Romanian version into English. The level of equivalence of both translated versions regarding the instrument’s original version was qualitatively assessed by the main author. Thereupon, a new group of 2 researchers inspected the content of every BRSQ item in the Romanian version from a qualitatively approach to guarantee that each measured the target psychological variable. Lastly, a pilot study was developed to confirm the correct understanding of the totality of items, administrating the BRSQ to 11 athletes. They stated the lack of problems in the understanding of the content of the 24 items. Altogether, these results provided validity evidence based on the BRSQ’s content.

### Data analysis

To provide validity evidence based on the BRSQ’s internal structure, a series of CFA and two invariance analyses were run. CFA were performed using the maximum likelihood method together with the 5000-resampling bootstrapping technique, due to the violation of the multivariate normality assumption (a Mardia’s coefficient = 239.31, *p* < .001) (Kline, 2016). The goodness of fit was assessed by several fit indexes: coefficient between *χ*^2^ and degree of freedom (*χ*^2^/df), comparative fit index (CFI), Tucker–Lewis index (TLI), incremental fit index (IFI), standardized root mean square residual (SRMR), root mean square error of approximation (RMSEA) with its confidence interval at 90% (90%CI) and Akaike information criterion (AIC). The *χ*^2^/*df* coefficient shows a good fit with values as high as 3, while values up to 5 represent an acceptable fit (Kline, 2016). CFI, TLI and IFI are excellent with values above .95, while they are acceptable when values are equal to .90 or greater (Kline, 2016). SRMR and RMSEA are indicative of a good fit to data with values less than .060, while values below .080 state an acceptable fit to data (Kline, 2016). AIC is a measure of parsimony used in the comparison of models, indicating that the model with the smallest value would be the most parsimonious and, hence, preferable (Kline, 2016). Standardized regression weights are acceptable when values are above .50 (Hair et al., 2018).

To examine measurement invariance across age and sport, and following the methodological approach by Kline (2016), four progressively constrained models were tested: configural invariance (no constraints), metric invariance (constraints in item factor loading), strong invariance (constraints in item factor loading and intercept, simultaneously) and strict invariance (constraints in item factor loading, intercept and error variance, simultaneously). Changes as high as .010 in CFI values paired with differences below .015 in RMSEA values between each two progressively constrained models would be indicative of the instrument’s invariant character (Kline, 2016). Regarding the age invariance analysis, the groups were created using median. Particularly, the first group (*i.e.,* younger athletes) was made up of 310 athletes aged between 18 and 21 years (*M*_*age*_ = 19.54, *SD*_*age*_ = 1.64), while the second group (*i.e.,* older athletes) included 286 athletes aged between 22 and 52 years (*M*_*age*_ = 27.64, *SD*_*age*_ = 5.02).

To examine the BRSQ’s convergent validity, average variance extracted (AVE) was estimated, which is appropriate with values as low as .50 (Hair et al., 2018). Given the concern reported in previous studies about the instrument’s discriminant validity (Lonsdale, Hodge & Rose, 2008; Holland et al., 2010), four criteria were used: (a) heterotrait-monotrait (HTMT) ratio of correlations (Henseler, Ringle & Sarstedt, 2015), which supports the discrimination among factors with values as high as .90 (Henseler, Ringle & Sarstedt, 2015; Henseler, Ringle & Sarstedt, 2015)); (b) if the interfactor correlation is less than unity by 1.96 times its standard error (Bagozzi & Kimmel, 1995); (c) correlations among latent factors, indicating that values less than .90 are representative of an acceptable conceptual discrimination among variables (Kline, 2016); and (d) confidence intervals at 95% (95% CI) of the correlation in question does not include 1.00 (Anderson & Gerbing, 1988). To inspect reliability of primary-order factors, Cronbach’s alpha (*α*), McDonald’s omega (*ω*) and Raykov’s composite reliability (*ρ*) coefficients were, respectively, calculated. Additionally, to examine the construct reliability of hierarchical factors, coefficient *H* was computed. Every coefficient shows a good level of reliability with values over .70 (Hair et al., 2018).

To gather criterion validity evidence, two linear regression analyses were conducted. In both analyses, the six types of behavioral regulation were introduced as independent variables, resilience and burnout were considered as dependent variables, while age and type of sport were covariates. Descriptive statistics was computed for every variable under study, while the univariate normality assumption was inspected by standardized values for univariate skewness and kurtosis coefficients. Thus, standardized values as high as 1.96 are representative of a normal data distribution (Hair et al., 2018). Finally, independent *t*-tests were run to examine differences by age and sport in the target variables. Data were statically processed using SPSS and AMOS statistical software, version 25.

## Results

### Preliminary results

The Brief Resilience Scale in its Romanian adaptation revealed adequate fit-indexes: *χ*^2^ (9, *N* = 596) = 43.66, *p* < .001, *χ*^2^/*df* = 4.85; CFI = .97, TLI = .93; IFI = .97, SRMR = .042; RMSEA = .079 (90% CI = .067–.089). Suitable values were also obtained for reliability (*α* = .83, *ρ* = .83) and convergent validity (AVE = .54). On the other hand, the Romanian version of the Athlete Burnout Measure displayed acceptable fit indexes: *χ*^2^ (87, *N* = 596) = 405.44, *p* < .001, *χ*^2^/*df* = 4.66; CFI = .94, TLI = .91; IFI = .94, SRMR = .056; RMSEA = .080 (90% CI =.068–.093). The three factors showed a good level of reliability (*α* = .79, *ρ* = .79; *α* = .77, *ρ* = .77; *α* = .82, *ρ* = .82) and convergent validity (AVE values of .51, .60, and .61).

### Main results

#### Confirmatory factor analyses

Table 1 shows the results obtained by CFA for each factor model tested for the BRSQ. Specifically, while none of the alternative models had a minimally acceptable fit to the data; the six-factor correlated model displayed an appropriate fit with the observed data, as well as the lowest AIC value. This suggested that the six-factor correlated model had to be used for the remaining analyses by obtaining the best psychometric performance.

**Table 1:** Goodness-of-fit measures for all BRSQ models tested.

Models	*χ*^2^(*df*)	*χ*^2^/*df*	CFI	TLI	IFI	SRMR	RMSEA(90% CI)	AIC
Primary-order models								
2-factor correlated model (SDM, NSDM)	1622.44(251)	6.46	.83	.81	.83	.073	.096(.091–.100)	1720.44
3-factor correlated model (IM, EM, AMOT)	2969.36(249)	11.93	.66	.63	.66	.159	.136(.131–.140)	3071.36
3-factor correlated model (AM, CM, AMOT)	1241.08(249)	4.99	.88	.86	.88	.059	.082(.077–.086)	1344.08
4-factor correlated model (IM, AEM, CEM, AMOT)	1135.16(246)	4.61	.89	.88	.89	.057	.078 .073–.083)	1243.16
5-factor correlated model (IM, InR+IdR, ItR, ER, AMOT)	1076.35(242)	4.45	.90	.88	.90	.055	.076(.072–.081)	1192.35
5-factor correlated model (IM, InR, IdR, IntR+ER, AMOT)	1054.92(242)	4.36	.90	.89	.90	.054	.075(.071–.080)	1170.92
6-factor correlated model (six motivational forms)	857.96(237)	3.62	.93	.91	.93	.050	.066(.062–.071)	979.63
Hierarchical models								
3-factor model (IM, EM, AMOT)	1799.95(245)	7.35	.81	.78	.81	.145	.103(.099–.108)	1909.95
3-factor model (AM, CM, AMOT)	873.55(244)	3.58	.92	.91	.92	.053	.066(.061–.071)	981.38

**Notes.**

SDM Self-determined motivation NSDM Non-self-determined motivation EM Extrinsic motivation AMOT Amotivation InR+IdR merged from the integrated and identified regulations factors AM Autonomous motivation CM Controlled motivation AEM Autonomous extrinsic motivation CEM Controlled extrinsic motivation

**Figure 1:**
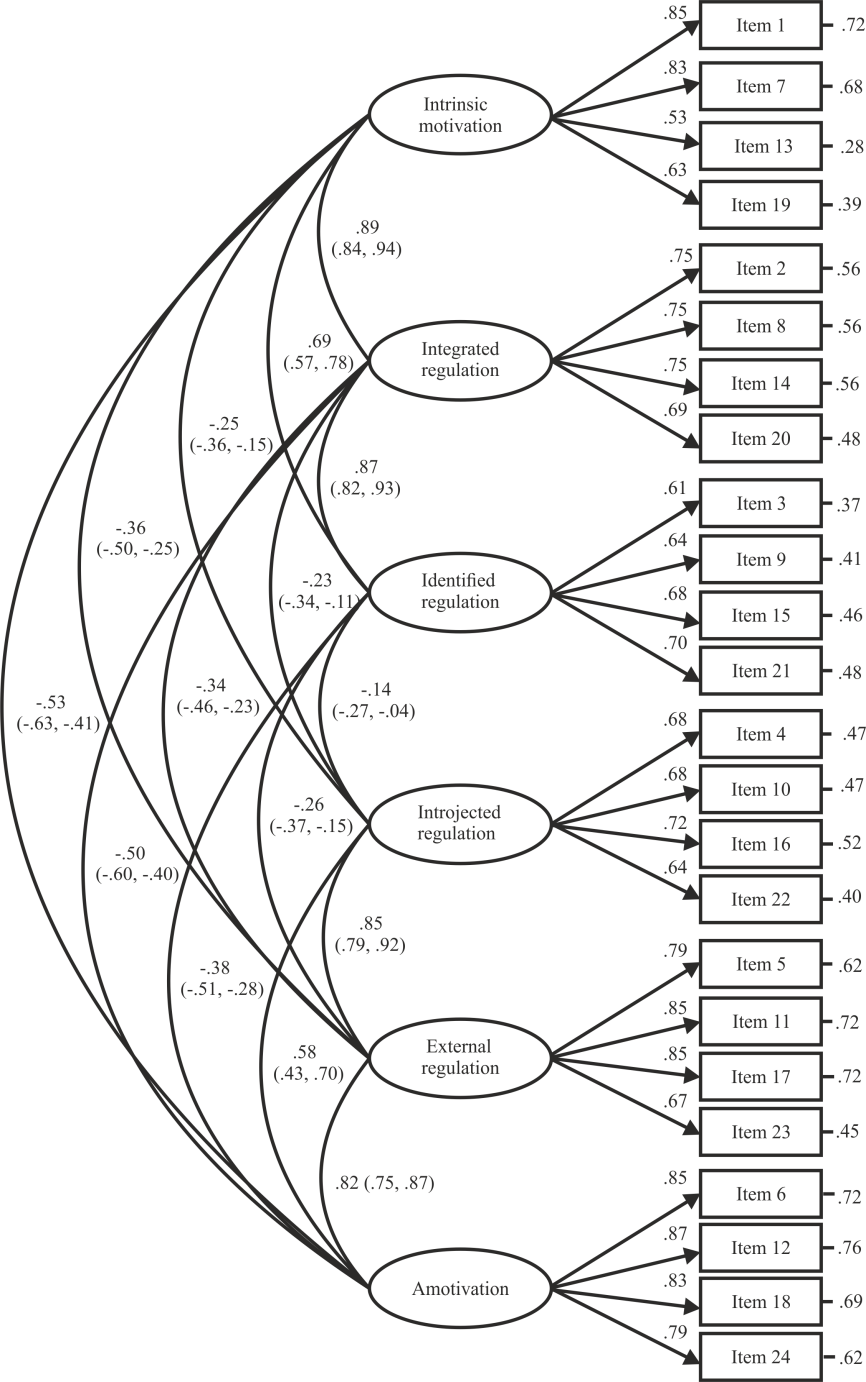
Confirmatory factor analysis for the Romanian version of the Behavioral Regulation in Sport Questionnaire. Note: The ellipses represent the latent factors, while the rectangles represent the different items. Numbers in parentheses show the standard error estimated by bootstrapping.

Figure 1 displays standardized regression weights, correlations among latent factors and squared multiple correlations for the six-factor correlated model. Standardized regression weights were between .53 and .87, each reaching the level of statistical significance (*p* < .001). Correlations among latent factors ranged from −.53 to .89, configuring a *simplex* structure with stronger and positive correlations among adjacent behavioral regulations, weaker correlations among more distal regulations, and negative correlations among ends.

On the other hand, Table 1 also shows the results from the CFA for each hierarchical model tested. It should be underscored that the three-factor model composed by autonomous motivation, controlled motivation and amotivation was the only that obtained an acceptable fit with the data. In this model, factor loadings were of .88 and .90 for intrinsic motivation, integrated regulation and identified regulation in the hierarchical autonomous motivation factor; while introjected regulation and external regulation obtained a factor loading of .88 and .90 in controlled motivation. Correlations among hierarchical factors were: *r* = −.34 between autonomous motivation and controlled motivation, *r* = −.51 between autonomous motivation and amotivation, and *r* = .80 between controlled motivation and amotivation.

#### Invariance analysis

Table 2 shows the absence of changes over .010 in CFI values accompanied by differences lower than .015 in RMSEA values in the successive constrained models for both multi-group analyses. Therefore, the null hypothesis of invariance across age and type of sport could not be rejected, respectively.

**Table 2:** Multi-group analysis of invariance.

	*χ*^2^(*df*)	CFI	RMSEA(90% CI)	MC	Δ*χ*^2^(Δ*df*)	ΔCFI	ΔRMSEA
Invariance across Age							
1. Configural invariance	1331.07(474)	.903	.053(.049–.057)	–	–	–	–
2. Metric invariance	1357.97(492)	.902	.052(.049–.056)	2 *vs* 1	26.90(18)	−.001	−.001
3. Strong invariance	1436.88(516)	.897	.053(.049–.056)	3 *vs* 2	78.91(24)	−.005	.001
4. Strict invariance	1589.65(540)	.887	.057(.053–.060)	4 *vs* 3	152.77(24)	−.010	.004
Invariance across Sport							
1. Configural invariance	1324.87(474)	.903	.053(.049–.056)	–	–	–	–
2. Metric invariance	1352.45(492)	.902	.052(.048–.055)	2 *vs* 1	28.58(18)	−.001	−.001
3. Strong invariance	1427.47(516)	.895	.052(.049–.056)	3 *vs* 2	75.76(24)	−.007	.000
4. Strict invariance	1572.51(540)	.886	.055(.051–.058)	4 *vs* 3	144.43(24)	−.009	.003

**Notes.**

MC Models comparison *vs* versus

#### Convergent and discriminant validity, and reliability analysis

Table 3 displays AVE values between .50 and .70, underpinning the instrument’s convergent validity. Table 2 also shows HTMT values as high as .88 among the six latent factors together scores for correlations of each behavioral regulation lower than 1.00 by a value 1.96 times its standard error, as well as interfactor correlations between -.53 and .89 and its 95% CIs that did not exceed the unity in all cases. These four results endorsed the BRSQ’s discriminant validity. On the other hand, Table 2 reflects acceptable reliability scores for the six primary-order factors with Cronbach’s alpha from .75 to .90, McDonald’s omega from .84 to .91, and Raykov’s coefficient from .75 to .90. For the hierarchical factors, a coefficient *H* was obtained of .93 for autonomous motivation and .92 for controlled motivation.

**Table 3:** Reliability coefficients, convergent and discriminant validity for the BRSQ.

	*α*	*ω*	*ρ*	AVE	1	2	3	4	5	6
1. Intrinsic motivation	.85	.87	.85	.52	–	.89(.84, .94) [0.894]	.69(.57, .78)[0.901]	−.25(−.36, −.15) [0.888]	−.36(−.50, −.25) [0.892]	−.53(−.63, −.41) [0.891]
2. Integrated regulation	.82	.90	.83	.54	.85	–	.87(.82, .93) [0.890]	−.23(−.34, −.11) [0.890]	−.34(−.46, −.23) [0.896]	−.50(−.60, −.40) [0.865]
3. Identified regulation	.75	.84	.75	.50	.68	.83	–	−.14(−.27, −.04) [0.904]	−.26(−.37, −.15) [0.989]	−.38(−.51, −.28) [0.867]
4. Introjected regulation	.78	.88	.78	.51	−.21	−.21	−.13	–	.85(.79, .92) [0.855]	.58(.43, 70) [0.737]
5. External regulation	.85	.90	.87	.62	−.32	-.30	−.24	.83	–	.82(.74, .87) [0.837]
6. Amotivation	.90	.91	.90	.70	−.50	−.46	−.36	.68	.88	–

**Notes.**

Numbers above diagonal represent correlations among latent factors with its 95% confidence interval in parenthesis and values in terms of unity by 1.96 times the standard error of the correlation in square brackets. Numbers below diagonal represent heterotrait-monotrait ratio of correlations.

#### Linear regression analysis

Table 4 shows that, after controlling for age and sport, intrinsic motivation (*β* =.21, *p* < .001), integrated regulation (*β* =.31, *p* < .001) and identified regulation (*β* =.13, *p* = .032) positively and significantly predicted resilience; while introjected regulation (*β* =.06, *p* = .036), external regulation (*β* =.14, *p* = .005) and amotivation (*β* =.63, *p* < .001) positively and significantly predicted burnout. The total variance explained was 25% for resilience and 52% for burnout.

**Table 4:** Linear regression analysis predicting resilience and burnout from behavioral regulation in athletes.

	Resilience							Burnout						
	*B*(*SE*)	*β*	*p-*value	*t*	Tolerance	VIF	*R* ^2^	*B*(*SE*)	*β*	*p-*value	*t*	Tolerance	VIF	*R* ^2^
(constant)	1.55(0.31)	–	<.001	8.06	–	–	.25	2.42(0.22)	–	<.001	10.79	–	–	.52
Age	0.02(0.01)	.14	<.001	3.70	0.95	1.05		−0.01(0.01)	−.01	.799	−0.26	0.95	1.05	
Sport	0.06(0.06)	−0.01	.709	−0.37	0.89	1.11		0.01(0.02)	.01	.790	0.27	0.89	1.11	
Intrinsic motivation	0.12(0.03)	.21	<.001	3.80	0.49	2.04		−0.02(0.03)	−.03	.480	−0.71	0.49	2.04	
Integrated regulation	0.28(0.06)	.31	<.001	4.98	0.34	2.93		−0.01(0.03)	−.01	.787	−0.27	0.34	2.93	
Identified regulation	0.11(0.05)	.11	.032	2.15	0.49	2.03		−0.03(0.04)	−.04	.377	−0.88	0.49	2.03	
Introjected regulation	−0.01(0.03)	−.01	.791	−0.27	0.46	2.17		0.09(0.04)	.06	.036	2.10	0.46	2.17	
External regulation	−0.02(0.04)	−.03	.585	−0.55	0.37	2.71		0.11(0.04)	.14	.005	2.79	0.37	2.71	
Amotivation	−0.03(0.05)	−.03	.592	−0.54	0.41	2.46		0.33(0.02)	.63	<.001	13.91	0.41	2.46	

**Notes.**

VIF Variance Inflation Factor

#### Descriptive statistics and differences by age and sport

Table 5 shows mean scores over mid-point of the respective measurement scale in intrinsic motivation, integrated regulation, identified regulation and resilience among athletes. Alternatively, there were mean values below mid-point of the measurement scale for introjected regulation, external regulation, amotivation and burnout. Standardized values ranged from −1.76 to 1.94 for skewness and from −0.22 to 1.71 for kurtosis, supporting the univariate normality assumption. Independent *t*-tests displayed that while older athletes higher scored than younger athletes in intrinsic motivation, integrated and identified regulation, and resilience; younger athletes obtained higher values of amotivation and burnout. Similarly, team sports athletes obtained higher scores in intrinsic motivation, integrated regulation and resilience, while individual sports athletes scored higher in amotivation.

**Table 5:** Descriptive statistics and differences by age and sport.

	Total sample				Younger athletes	Older athletes	*t*-tests			Individual sports	Team sports	*t*-tests		
	Range	*M*(*SD*)	*γ*_1_	*γ*_2_	*M*(*SD*)	*M*(*SD*)	*t(df)*	*p*-value	*d*	*M*(*SD*)	*M*(*SD*)	*t(df)*	*p*-value	*d*
Intrinsic motivation	1–7	6.33(0.87)	−1.76	1.61	6.20(0.98)	6.47(0.71)	3.78(594)	<.001	0.32	6.08(0.99)	6.54(0.68)	6.72(594)	<.001	0.54
Integrated regulation	1–7	6.30(0.89)	−1.66	1.71	6.16(0.98)	6.53(0.73)	5.10(594)	<.001	0.43	6.15(1.02)	6.49(0.73)	4.64(594)	<.001	0.38
Identified regulation	1–7	6.32(0.84)	−1.52	1.44	6.21(0.90)	6.44(0.76)	3.32(594)	<.001	0.28	6.29(0.85)	6.35(0.84)	0.86(594)	.390	0.07
Introjected regulation	1–7	2.38(1.46)	1.09	0.39	2.49(1.40)	2.26(1.52)	1.93(594)	.054	0.15	2.45(1.45)	2.32(1.48)	1.09(594)	.276	0.08
External regulation	1–7	1.85(1.24)	1.94	1.67	1.94(1.24)	1.76(1.23)	1.80(594)	.072	0.14	1.96(1.29)	1.76(1.18)	1.98(594)	.049	0.16
Amotivation	1–7	1.99(1.39)	1.72	1.25	2.10(1.42)	1.87(1.35)	2.04(594)	.042	0.17	2.23(1.51)	1.79(1.26)	3.86(594)	<.001	0.32
Resilience	1–5	3.63(0.76)	−0.13	−0.22	3.47(0.76)	3.80(0.72)	5.47(594)	<.001	0.45	3.55(0.82)	3.69(0.70)	2.28(594)	.023	0.33
Burnout	1–5	2.12(0.73)	0.63	−0.15	2.21(0.72)	2.03(0.73)	2.95(594)	.003	0.25	2.18(0.78)	2.08(0.69)	1.69(594)	.093	0.14

**Notes.**

γ_1_ Standardized coefficient for skewness γ_2_ Standardized coefficient for kurtosis *df* degree of freedom *d* Cohen’s d effect size measure

## Discussion

The objective of this research was to gather validity and reliability evidence for the use of the BRSQ in Romanian professional athletes. The results were consistent the hypotheses raised in this study, and, therefore, provided a strong psychometric support for the BRSQ as a valid and reliable measure of motivation from the SDT framework (Ryan & Deci, 2020) in the Romanian sport domain.

### Confirmatory factor analyses

Similar to the results reported in previous research that compared the original six-factor correlated model with alternative correlated models of the BRSQ (Lonsdale et al., 2014; Hancox et al., 2015; Monteiro, Moutão & Cid, 2018; Rodrigues et al., 2020), the original six-factor model obtained the best psychometric performance. Indeed, the goodness-of-fit measures were similar to the distinct adapted BRSQ versions to other social-cultural contexts (Assor, Vansteenkiste & Kaplan, 2009; Holland et al., 2010; Viladrich, Torregrosa & Cruz, 2011; Çetinkaya & Mutluer, 2018). Furthermore, all standardized regression weights were higher than .50, stating the each of them adequately represented the factor theoretically intended. Correlations among latent factors displayed a *simplex* structure with stronger and positive correlations among adjacent behavioral regulations, weaker correlations among more distal behavioral regulations, and negative correlations among extremes. These findings were aligned with previous studies focused on the BRSQ (Assor, Vansteenkiste & Kaplan, 2009; Viladrich, Torregrosa & Cruz, 2011; Hancox et al., 2015; Cece et al., 2019) and they, in turn, gathered evidence to psychometrically underpin the existence of the self-determination *continuum* advocated by SDT (Ryan & Deci, 2020).

On the other hand, the results from CFA also underpinned a hierarchical three-factor model encompassing autonomous motivation, controlled motivation and amotivation. This suggests that intrinsic motivation, integrated and identified regulation could be representing autonomous motivation, while introjected and external regulation could be representing controlled motivation. Indeed, these findings provided support for three general types of motivation in accordance with the SDT paradigm (Ryan & Deci, 2020; Ryan et al., 2021) considering their quality. Furthermore, these results are of a great methodological utility when researchers want to study antecedents and outcomes of motivation into complex structural models.

### Invariance

The results derived from the two multi-group analyses provided evidence underpinning the measurement invariance across age and sport for the 24-item, six-factor correlated model, in line with the original version of the instrument (Lonsdale, Hodge & Rose, 2008) and with the different adaptations to other contexts (Hancox et al., 2015; Stenling et al., 2018; Cece et al., 2019; Monteiro et al., 2019). Particularly, these findings are of great practical utility by allowing us to recommend the use of the BRSQ to more deeply study the possible differences in the level of each behavioral regulation in athletes with different age and type of sport practiced (*i.e.,* individual or team) in the Romanian domain.

### Convergent and discriminant validity, and reliability

With respect to the BRSQ convergent validity, our results showed AVE scores above .50 in the six factors comprising it, indicating that every item was strongly related to the motivational factor under measurement. Our findings, although they contrasted with marginal values manifested by Monteiro, Moutão & Cid (2018), were similar to those obtained in previous studies (Lonsdale, Hodge & Rose, 2008; Holland et al., 2010; Viladrich, Torregrosa & Cruz, 2011; Lonsdale et al., 2014; Monteiro et al., 2019). Concerning the instrument’s discriminant validity, evidence was met in support of an appropriate discrimination among the six types of behavioral regulations described by SDT (Ryan & Deci, 2020) and measured in the BRSQ (Lonsdale, Hodge & Rose, 2008). In line with previous research on the BRSQ (Lonsdale, Hodge & Rose, 2008; Holland et al., 2010; Monteiro, Moutão & Cid, 2018), high correlations in this study were also found between intrinsic motivation and integrated regulation (*r* = .89), between integrated and identified regulation (*r* = .87), and between introjected and external regulation (*r* = .85), although they did not suppose a discriminant validity problem by not exceeding .90 (Kline, 2016), not including the unity the 95% CI of each correlation and by being its score less than the unity by 1.96 times its standard error. In addition, it is important to underscore that the estimation of HTMT ratio of correlations (Henseler, Ringle & Sarstedt, 2015) with scores as high as .88 gathered new evidence endorsing the BRSQ discriminant validity. On the other hand, similar to the results reported both in the original version (Lonsdale, Hodge & Rose, 2008) and the different adaptations of the instrument (Assor, Vansteenkiste & Kaplan, 2009; Holland et al., 2010; Viladrich, Torregrosa & Cruz, 2011; Hancox et al., 2015; Çetinkaya & Mutluer, 2018; Monteiro, Moutão & Cid, 2018), a good level of reliability was obtained for each primary-order factor comprising the BRSQ with values of Cronbach’s alpha, McDonald’s omega and Raykov’s coefficient greater than .70. Also, it should be underlined that this is the first study that provided construct reliability evidence for the hierarchical factors considered in the BRSQ.

### Criterion validity

The results of the two linear regression analyses were consistent with the SDT assumptions (Ryan & Deci, 2020) and previous studies (Li et al., 2013; Clancy et al., 2016; Trigueros et al., 2020), supporting, therefore, criterion validity of the BRSQ. As expected, the three autonomous forms of motivation (*i.e.,* intrinsic motivation, integrated regulation and identified regulation) positively predicted resilience among professional athletes. A plausible explanation would rest on the fact that athletes tend to optimally develop a stronger sense of adaptation, resistance and recovery from stressing events when they engage in their sport for a combination of reasons based on enjoyment and seeking out optimal challenges, incorporation of their sport into their identity and the recognition of the its benefits. Thus, fostering the autonomous forms of motivation among athletes seems to be a promising strategy to optimally develop their resilience. The results also displayed a positive prediction effect of the two controlled forms of motivation (*i.e.,* introjected and external regulation) and, mainly, amotivation on burnout in professional athletes. This may be explained because athletes who participate in their sport guided by externally (*e.g.*, winning a tournament) and internally (*e.g.*, to gain self-esteem) controlled reasons, and perceptions of incompetence and a passive engagement, they will be prone to experience a reduced sense of accomplishment, emotional and physical exhaustion and devaluation when they failure to their set sports goals.

### Limitations

Given the complexity of the cognitive processes, validation of any measure of psychological variables should be understood as an ongoing process over time. Therefore, a series of limitations should be taken into account for the present study. Firstly, the purposive sampling method used in this research does not allow us to generalize the obtained results to the general population. Thus, new research could take into account more heterogeneous samples of athletes depending on their previous sports experience, performance level, gender, injuries, religion or ethical background. Secondly, the adoption of a cross-sectional design has not permitted to establish casual-effect relationships among the variables under study. Hence, it is not possible to ascertain if behavioral regulation is an antecedent of resilience and burnout in sport or vice-versa. Additional longitudinal studies are therefore needed to examine this issue.

### Practical Implications

The adaptation of the BRSQ to the Romanian sport domain allows us to assess athletes’ perceptions of the six types of behavioral regulation in this setting, implying a set of implications for practice. Methodologically, the Romanian version of the BRSQ constitutes the first well-validated measure of behavioral regulation toward sport under the SDT framework in Romania. Theoretically, our results gather a consistent body of evidence in support of the self-determination *continuum* advocated by SDT. In addition, the assessment of the quality of motivation might contribute to provide a better insight into the Romanian athletes’ motivational processes involved in sport. Practically, these results open the manner for implementing potentially effective interventions, positing that increased athletes’ levels of the autonomous forms of motivation may enhance their performance-related outcomes. Additionally, the Romanian version of the BRSQ could be used both to analyze changes in athletes’ behavioral regulation throughout a single season, and to examine their motivational trajectories in the course of their sports careers. Furthermore, this tool will allow coaches to conducted a more accurate detection of those athletes who might be at a motivational risk at any point of the season. Taken together, this information will help coaches implement more adapted motivational strategies in order to effectively develop adaptive motivational patterns among athletes.

## Conclusions

The present research shows that the BRSQ can be utilized to assess both the six types of behavioral regulation and the three general qualities of motivation toward sport with Romanian athletes. Specifically, this study gathered a substantial basis of evidence to support both validity (*i.e.,* internal structure, convergent, discriminant, and criterion) and reliability of this instrument. Thus, it is noteworthy that now the scale is available as a Romanian measurement instrument of motivation underlying to the SDT framework in the sport domain, as it contributed to filling an existing gap until now in Romania.

## Supplemental Information

10.7717/peerj.12803/supp-1 Supplemental Information 1 Raw data used for the validation study

10.7717/peerj.12803/supp-2 Supplemental Information 2 Romanian version of the Behavioral Regulation in Sport Questionnaire

## Additional Information and Declarations

### Data Availability

The following information was supplied regarding data availability: The raw measurement are available in the Supplementary Files.
