# Supplementary material for: Validity and reliability evidence for the Behavioral Regulation in Sport Questionnaire with Romanian professional athletes
Source: PeerJ. 2022 Jan 11;10:e12803. doi: 10.7717/peerj.12803 (PMC8759356; doi:10.7717/peerj.12803)
Supplement: Supplemental Information 2 [file peerj-10-12803-s002.docx]

**Appendix A**

*Romanian version of the Behavioral Regulation in Sport Questionnaire*

| Intrinsic motivation |
| --- |
| 1. Practic sportul meu preferat pentru că simt bucurie  7. Practic sportul meu preferat pentru că îmi place  13. Practic sportul meu preferat pentru că e distractive  19. Practic sportul meu preferat pentru că mi se pare plăcut |
| Integrated regulation |
| 2. Practic sportul meu preferat pentru ca mă reprezintă  8. Practic sportul meu preferat pentru că este o oportunitate de a fi eu însumi/însămi  14. Practic sportul meu preferat pentru că ce fac eu în sport este o expresie a identității mele  20. Practic sportul meu preferat pentru că îmi permite să trăiesc într-un mod care este conform cu valorile mele |
| Identified regulation |
| 3. Practic sportul meu preferat pentru ca beneficiile sportului sunt importante pentru mine  9. Practic sportul meu preferat pentru că mă învață autodisciplina  15. Practic sportul meu preferat pentru că valorizez beneficiile sportului pe care îl practice  21. Practic sportul meu preferat pentru că este o metodă bună de a învăța lucruri noi, care ar putea să mă ajute în viață |
| Introjected regulation |
| 4. Practic sportul meu preferat pentru că mi-ar fi rușine dacă aș renunța  10. Practic sportul meu preferat pentru că m-aș simți un ratat/o ratată dacă aș renunța  16. Practic sportul meu preferat pentru că mă simt obligat(ă) să continui  22. Practic sportul meu preferat pentru că mă voi simți vinovat dacă voi renunța la el |
| External regulation |
| 5. Practic sportul meu preferat pentru că, dacă nu o fac, alți oameni nu vor fi mulțumiți de mine  11. Practic sportul meu preferat pentru că simt presiune din partea altor persoane ca eu să practic acest sport  17. Practic sportul meu preferat pentru că alte persoane mă forțează să joc  23. Practic sportul meu preferat pentru a îi mulțumi pe oamenii care își doresc ca eu să fac acest lucru |
| Amotivation |
| 6. Practic sportul meu preferat, dar totuși mă întreb pentru ce motiv îl practice  12. Practic sportul meu preferat, dar mă întreb de ce mai continui acest lucru  18. Practic sportul meu preferat, dar motivele pentru care o fac nu-mi mai sunt clare  24. Practic sportul meu preferat, dar mă întreb de ce mă chinui așa mult |
